# High linear energy transfer carbon-ion irradiation upregulates PD-L1 expression more significantly than X-rays in human osteosarcoma U2OS cells

**DOI:** 10.1093/jrr/rrab050

**Published:** 2021-07-01

**Authors:** Tiara Bunga Mayang Permata, Hiro Sato, Wenchao Gu, Sangeeta Kakoti, Yuki Uchihara, Yukihiko Yoshimatsu, Itaru Sato, Reona Kato, Motohiro Yamauchi, Keiji Suzuki, Takahiro Oike, Yoshito Tsushima, Soehartati Gondhowiardjo, Tatsuya Ohno, Takaaki Yasuhara, Atsushi Shibata

**Affiliations:** Department of Radiation Oncology, Gunma University, Maebashi, Gunma, 371-8511, Japan; Department of Radiation Oncology, Faculty of Medicine Universitas Indonesia – Dr. Cipto Mangunkusumo Hospital, Jakarta, 10430, Indonesia; Department of Radiation Oncology, Gunma University, Maebashi, Gunma, 371-8511, Japan; Gunma University Heavy Ion Medical Center, Maebashi, Gunma, 371-8511, Japan; Gunma University Initiative for Advanced Research (GIAR), Gunma University, Maebashi, Gunma, 371-8511, Japan; Department of Diagnostic Radiology and Nuclear Medicine, Gunma University Graduate School of Medicine, Maebashi, Gunma, 371-8511, Japan; Department of Radiation Oncology, Gunma University, Maebashi, Gunma, 371-8511, Japan; Gunma University Initiative for Advanced Research (GIAR), Gunma University, Maebashi, Gunma, 371-8511, Japan; Gunma University Initiative for Advanced Research (GIAR), Gunma University, Maebashi, Gunma, 371-8511, Japan; Gunma University Initiative for Advanced Research (GIAR), Gunma University, Maebashi, Gunma, 371-8511, Japan; Department of Diagnostic Radiology and Nuclear Medicine, Gunma University Graduate School of Medicine, Maebashi, Gunma, 371-8511, Japan; Gunma University Initiative for Advanced Research (GIAR), Gunma University, Maebashi, Gunma, 371-8511, Japan; Laboratory of Molecular Radiology, Center for Disease Biology and Integrative Medicine, Graduate School of Medicine, The University of Tokyo, Bunkyo-ku, Tokyo, 113-8655, Japan; Department of Radiation Biology and Protection, Atomic Bomb Disease Institute, Nagasaki University, Nagasaki, 852-8523, Japan; Department of Radiation Medical Science, Atomic Bomb Disease Institute, Nagasaki University, Nagasaki, 852-8523, Japan; Department of Radiation Oncology, Gunma University, Maebashi, Gunma, 371-8511, Japan; Gunma University Initiative for Advanced Research (GIAR), Gunma University, Maebashi, Gunma, 371-8511, Japan; Department of Radiation Oncology, Faculty of Medicine Universitas Indonesia – Dr. Cipto Mangunkusumo Hospital, Jakarta, 10430, Indonesia; Department of Radiation Oncology, Gunma University, Maebashi, Gunma, 371-8511, Japan; Gunma University Heavy Ion Medical Center, Maebashi, Gunma, 371-8511, Japan; Laboratory of Molecular Radiology, Center for Disease Biology and Integrative Medicine, Graduate School of Medicine, The University of Tokyo, Bunkyo-ku, Tokyo, 113-8655, Japan; Gunma University Initiative for Advanced Research (GIAR), Gunma University, Maebashi, Gunma, 371-8511, Japan

**Keywords:** anti-PD-1/PD-L1 therapy, high linear energy transfer (LET) carbon-ion therapy, PD-L1 expression, DNA damage response

## Abstract

Programmed death ligand 1 (PD-L1) expression on the surface of cancer cells affects the efficacy of anti-PD-1/PD-L1 immune checkpoint therapy. However, the mechanism underlying PD-L1 expression in cancer cells is not fully understood, particularly after ionizing radiation (IR). Here, we examined the impact of high linear energy transfer (LET) carbon-ion irradiation on the expression of PD-L1 in human osteosarcoma U2OS cells. We found that the upregulation of PD-L1 expression after high LET carbon-ion irradiation was greater than that induced by X-rays at the same physical and relative biological effectiveness (RBE) dose, and that the upregulation of PD-L1 induced by high LET carbon-ion irradiation was predominantly dependent on ataxia telangiectasia and Rad3-related (ATR) kinase activity. Moreover, we showed that the downstream signaling, e.g. STAT1 phosphorylation and IRF1 expression, was upregulated to a greater extent after high LET carbon-ion irradiation than X-rays, and that IRF1 upregulation was also ATR dependent. Finally, to visualize PD-L1 molecules on the cell surface in 3D, we applied immunofluorescence-based super-resolution imaging. The three-dimensional structured illumination microscopy (3D-SIM) analyses revealed substantial increases in the number of presented PD-L1 molecules on the cell surface after high LET carbon-ion irradiation compared with X-ray irradiation.

## INTRODUCTION

Radiotherapy is one of the main pillars of cancer treatment, and recent technological developments have significantly improved its therapeutic efficacy. Among the advanced radiotherapy modalities, particle therapy has two significant advantages over photon-based therapy. First, carbon-ion irradiation, which is categorized as heavy-ion particle therapy, produces a 2–3-fold greater relative biological effectiveness (RBE) compared with conventional radiotherapy using X-rays or γ-irradiation [1–3]. Second, carbon-ion irradiation causes highly concentrated dose distributions, which allow intensive targeting of cancer cells so that damage to surrounding normal tissues is minimized because of the Bragg peak effect.

Immunotherapy using an immune checkpoint inhibitor is one of the most promising cancer therapies that have been licensed for the treatment of various tumors [4, 5]. Cytotoxic T lymphocytes express the PD-1 or CTLA-4 receptors, which bind to their ligands, programmed death 1 (PD-1) or CD80/CD86, respectively, expressed on the surfaces of antigen-presenting cells, e.g. cancer cells or dendritic cells. The interaction between PD-L1 and PD-1 or CD80/86 and CTLA-4 transduces a signal suppressing the antitumor activity of immune cells. Treatment with anti-PD-1/PD-L1 antibodies, which are defined as immune checkpoint inhibitors, blocks the interaction between PD-1 and PD-L1 and restores antitumor immunity. However, despite the elegant modes of action of immune checkpoint inhibitors, the frequency of high responders to this therapy remains low (<20%), and cases of complete remission are very few [5, 6]. To improve the efficacy of immune checkpoint inhibitor therapy in non-high-responder patients, anti-PD-1/PD-L1 antibody treatments are often combined with conventional cancer treatments [7, 8]. Recent evidence from clinical and preclinical studies shows that combinations of an anti-PD-1/PD-L1 antibody with conventional cancer therapies, such as radiotherapy and chemotherapy, are more effective than either treatment alone [9–12]. Among the conventional cancer therapies, carbon-ion radiotherapy is considered one of the best partners of anti-PD-1/PD-L1 treatment, because carbon-ion radiotherapy specifically targets solid tumors owing to the Bragg peak effect, whereas anti-PD-1/PD-L1 therapy is complementarily effective against metastases as well as solid tumors. Accordingly, recent reports suggest that anti-PD-L1 antibody treatments enhance antitumor efficacy in combination with carbon-ion radiotherapy, as assessed in a clinical trial as well as in a tumor mouse model [13, 14].

Previously, we demonstrated that DNA damage signaling after ionizing radiation (IR) upregulates PD-L1 expression on cancer cell surfaces [15, 16]; however, the manner in which PD-L1 expression is regulated after carbon-ion irradiation in the context of different linear energy transfer (LET) conditions remains unknown. In this study, we investigated the impact of high LET carbon-ion irradiation on PD-L1 expression in human osteosarcoma U2OS cells, which were used in our previous studies, as well as its underlying mechanisms.

## MATERIALS AND METHODS

### Cell culture, irradiation and drug treatment

The human osteosarcoma cancer cell line U2OS was obtained from ATCC® (HTB-96™). The U2OS cells were cultured in Eagle’s Minimum Essential Medium with 10% fetal calf serum. X-ray irradiation was performed using an MX-160Labo (160 kVp, 1.07 Gy/min, 3.00 mA; mediXtec, Japan). Cells were passaged by 1/10 or 1/5 dilution rate before cell confluency of 90–100% was achieved. At 30 min before irradiation, an ATR inhibitor (ATRi; VE821; Axon Medchem) or an ATM inhibitor (ATMi; KU55933; Merck Chemicals) was added at 10 μM. Prior to mono peak carbon-ion irradiation, media were removed from dishes. During mono peak carbon-ion irradiation, the dishes were covered with 8 μm Kapton polyimide film to avoid drying. Mono peak carbon-ion irradiation was performed at our Heavy Ion Medical Center at 290 MeV/n and with LET at 13, 20, 40 or 60 keV/μm.

### Analysis of cell surface PD-L1 expression by flow cytometry

After exposure to irradiation, cancer cells were incubated for 48 h and were then harvested for flow cytometry analysis. U2OS cells were washed with 1 mM EDTA/phosphate buffered saline (PBS) once, followed by incubation with 1 mM EDTA/PBS for 5 min. Cells were then harvested by pipetting in 1 mM EDTA/PBS without trypsin. Harvested cells were washed with 1 mM EDTA/PBS and stained with anti-PD-L1 antibodies for 20 min on ice. Dead cells were detected using propidium iodide (Sigma-Aldrich) and were excluded from analyses. Flow cytometry analysis was performed on an Attune NxT Flow Cytometer (Thermo Fisher Scientific). The median fluorescence intensity (MFI; PD-L1-to-isotype) was calculated as follows: MFI (PD-L1) − MFI (isotype control).

### Analyses of cell surface PD-L1 expression using a super-resolution 3D-SIM OMX microscope

At 48 h after IR, the cells on the coverslips were directly incubated with the primary antibody in the culture medium at 37°C in a CO_2_ incubator for 30 min, followed by fixation with a 3% paraformaldehyde-2% sucrose solution for 10 min. After three PBS washes, the cells were blocked with 2% bovine serum albumin (BSA)-PBS for 30 min at room temperature, followed by incubation with the secondary antibody (Alexa 488) for 30 min at 37°C. For 4′,6-diamidino-2-phenylindole, dihydrochloride (DAPI; Roche, Mannheim, Germany) staining, the cells were permeabilized with a 2% Triton X-PBS solution for 3 min, and the cells on the coverslips were mounted with Vectashield (Vector Laboratories, Burlingame, CA, USA).

Three-dimensional structured illumination microscopy (3D-SIM) was performed as described previously [17]. Briefly, the microscope system (DeltaVision OMX version 4, GE Healthcare UK Ltd) was equipped with 405 and 488 nm solid-state lasers. Optical z-sections were separated by 0.125 μm and 3D-SIM data were acquired using laser lines of 405 and 488 nm. The exposure times were typically between 60 and 80 minutes, and the power of each laser was adjusted to achieve optimal intensities (between 4000 and 15 000 counts) in raw images in the 15-bit dynamic range. To minimize photobleaching, exposures were performed at the lowest laser power possible. Multichannel imaging was achieved through sequential acquisitions of wavelengths using separate cameras. Raw 3D-SIM images were processed and reconstructed using the DeltaVision OMX SoftWoRx 6.1 software package (GE Healthcare).

**Fig. 1.**
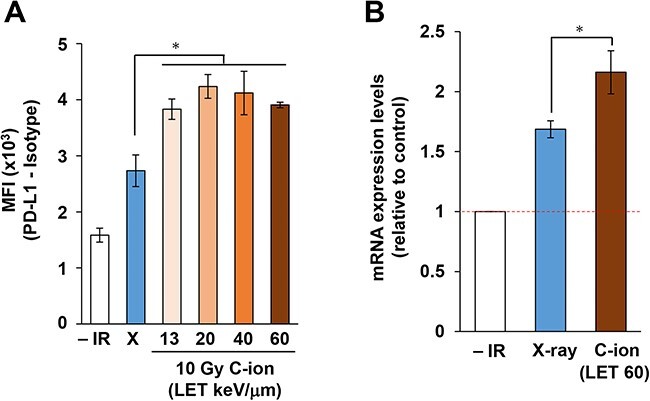
Carbon-ion irradiation upregulates PD-L1 expression more than does X-ray irradiation in U2OS cells. (A) Flow cytometry analyses of cell surface PD-L1 expression levels were performed in U2OS cells 48 h after 10 Gy X-ray or carbon-ion irradiation with LET at 13, 20, 40 and 60 keV/μm. The statistical significance of differences was examined by comparison with X-ray irradiated cells of the corresponding cell line using Bonferroni’s correction. ^*^P < 0.0125. (B) *PD-L1* mRNA expression levels in U2OS cells were examined 16 h after 10 Gy X-ray or carbon-ion irradiation with LET at 60 keV/μm. The statistical significance following Bonferroni’s correction is shown. ^*^P < 0.025.

The immunofluorescent PD-L1 signal was processed and analyzed using Imaris 8.1.2 (Bitplane, Zurich, Switzerland). The number of PD-L1 spots was detected using the Spots mode. To measure the volume of the PD-L1 signal, the 3D structure of the PD-L1 polygon rendering was generated using the Surface mode. In the Surface mode, the Background Subtraction function was selected, with the diameter of the largest Sphere set to 0.3 μm, for optimum viewing of PD-L1 expression. The volume of each PD-L1 polygon rendering was then recorded and analyzed. For the SIM analysis, at least 10 cells per experiment were analyzed in each condition. Similar results were obtained in two independent experiments.

**Fig. 2.**
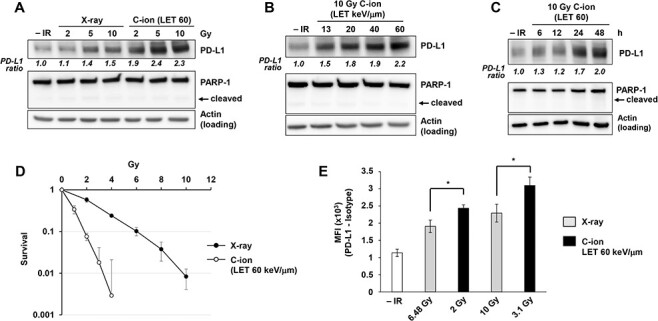
Carbon-ion irradiation upregulates PD-L1 protein expression in U2OS cells. (A) PD-L1 protein expression after carbon-ion irradiation with LET at 60 keV/μm was higher than that detected after X-ray irradiation. U2OS cells were harvested at 48 h after exposure to 2, 5, or 10 Gy X-ray or carbon-ion irradiation. Poly(ADP-ribose) polymerase (PARP-1) cleavage was examined to confirm that apoptosis was not induced in the analyzed cells. (B) PD-L1 upregulation in U2OS cells was examined 48 h after irradiation with 10 Gy of carbon-ions with LET at 13, 20, 40 and 60 keV/μm. (C) PD-L1 upregulation in U2OS cells was examined at the indicated time points after irradiation with 10 Gy of carbon-ions with LET at 60 keV/μm. (D) A colony formation assay was performed in U2OS cells to calculate RBE comparing X-ray and carbon-ion with LET at 60 keV/μm. (E) Flow cytometry analyses for cell-surface PD-L1 were performed in U2OS cells 48 h after 6.48 Gy or 10 Gy X-ray vs. 2 or 3.1 Gy carbon-ion irradiation with LET at 60 keV/μm, to set a similar RBE dose. The statistical significance following Bonferroni’s correction is shown. ^*^P < 0.0125. In A–D, the signal intensities of PD-L1 and actin were measured using ImageJ. The PD-L1 signal was normalized to that of actin; subsequently, the ratio of PD-L1 upregulation was normalized to that detected in non-irradiated cells.

### Immunoblotting

Immunoblotting was performed as described previously [15]. The lists of the antibodies used in the present study are provided in Supplementary Tables 1 and 2. The signal intensity was quantified using the ImageJ software.

**Fig. 3.**
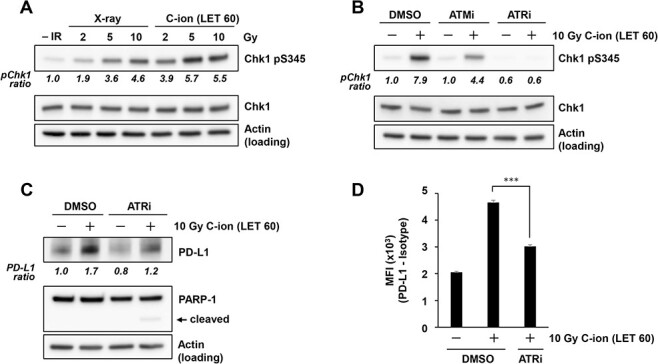
Carbon-ion irradiation upregulates PD-L1 expression in an ATR-dependent manner. (A) Chk1 phosphorylation at S345 in U2OS cells was examined at 2 h after 2, 5 or 10 Gy X-ray or carbon-ion irradiation with LET at 60 keV/μm. Because resection and Chk1 phosphorylation peak at ~2 h after irradiation, Chk1 phosphorylation was examined at 2 h after exposure to IR. (B) Chk1 phosphorylation at S345 in U2OS cells was examined in the presence of 10 μM ATM (KU55933) or 10 μM ATR inhibitor (VE821) at 2 h after irradiation with 10 Gy of carbon-ions with LET at 60 keV/μm. (C) PD-L1 expression in U2OS cells was examined in the presence of 10 μM ATR inhibitor (VE821) at 48 h after 10 Gy carbon-ion irradiation with LET at 60 keV/μm. (D) Cell surface PD-L1 expression in U2OS cells was examined in the presence of 10 μM ATR inhibitor (VE821) at 48 h after irradiation with 10 Gy of carbon-ions with LET at 60 keV/μm. The statistical significance following Bonferroni’s correction is shown. ^***^P < 0.0005. In A–B, the signal intensities of Chk1 pS345 and Chk1 were measured using ImageJ. The Chk1 pS345 signal was normalized to that of Chk1; subsequently, the ratio of Chk1 pS345 upregulation was normalized to that detected in non-irradiated cells. PD-L1 quantification in C was performed as described in Fig. 1.

### Quantification of mRNA expression levels by real-time PCR

Total RNA was extracted from cells using NucleoSpin RNA (MACHEREY-NAGEL) after X-ray or carbon-ion irradiation at 10 Gy. Total RNA was then reverse-transcribed into cDNA using a PrimeScript RT Reagent Kit (Perfect Real Time; TaKaRa), according to the manufacturer’s instructions. Quantitative PCR (qPCR) was performed using a StepOnePlus Real-Time PCR System (Life Technologies). Reactions (20 μL each) were prepared in duplicate in MicroAmp Fast Optical 96-Well Reaction Plates (Applied Biosystems). Reactions contained forward and reverse primers at 0.5 μM, the probe at 0.2 μM, 10 μl of TaqMan Universal PCR Master Mix (Applied Biosystems), and the cDNA template. Target-gene expression levels were normalized to those of *GAPDH* and were calculated using the 2^−ΔΔCt^ method. Thermal cycling for qPCR included an initial denaturation at 95°C for 10 min, followed by 45 cycles of denaturation at 95°C for 15 s and annealing and extension at 60°C for 1 min. The primers and probes used for qPCR were as follows:

*PD-L1* forward: 5′-GGAGATTAGATCCTGAGGAAAACCA-3′.

*PD-L1* reverse: 5′-AACGGAAGATGAATGTCAGTGCTA-3′.

*PD-L1* probe: 5′-AGATGGCTCCCAGAATTACCAAGTGAGTCC-3′.

*GAPDH* forward: 5′-CTCCTCTGACTTCAACAGCGA-3′.

*GAPDH* reverse: 5′-CCAAATTCGTTGTCATACCAGGA-3′.

*GAPDH* probe: 5′-ATGCCAGCCCCAGCGTCAAAGGT-3′.

### Statistical analysis

Differences between treatment groups were identified by Student’s two-tailed *t*-test or Mann–Whitney *U* tests using the GraphPad Prism 7.0 software or SigmaPlot 12.3. Significant values were corrected by Bonferroni’s correction if required.

## RESULTS

### PD-L1 is more significantly induced by high LET carbon-ion irradiation than by X-ray irradiation

X-ray irradiation upregulates PD-L1 expression in cancer cells [15]. To compare the induction of PD-L1 between X-ray and carbon-ion irradiation *in vitro*, we examined PD-L1 expression levels on the cell surface by flow cytometry. In this study, the U2OS cell line was selected to investigate PD-L1 expression, because the mechanism of DNA damage signal-dependent PD-L1 expression has been extensively investigated in these cells in our previous studies [15, 16] (N.B. the cyclic GMP-AMP synthase (cGAS)/stimulator of interferon genes (STING) pathway is downregulated in U2OS cells) [18]. Greater expression of PD-L1 on cell surfaces was observed after high LET carbon-ion irradiation compared with X-rays at the same physical dose of 10 Gy (Fig. 1a), although PD-L1 expression on the cell surface seemed to be saturated at LET of 20–60 keV/μm. Next, to investigate whether PD-L1 upregulation is induced at the transcriptional level after high LET carbon-ion irradiation, *PD-L1* mRNA expression was examined using qPCR. As in the flow cytometry experiments, the *PD-L1* mRNA was more significantly upregulated by carbon-ion irradiation compared with X-rays (Fig. 1b).

Next, to consolidate the finding that PD-L1 upregulation on the cell surface is associated with the induction of PD-L1 protein expression, we examined PD-L1 protein levels by immunoblotting in whole cell extracts. Upregulation of the PD-L1 protein was observed in cells exposed to X-rays at 5–10 Gy, whereas carbon-ion irradiation was as effective when used at 2 Gy (Fig. 2a). Importantly, carbon-ion irradiation at 5–10 Gy induced higher expression levels of PD-L1 than those detected after the delivery of the same physical dose of X-rays (Fig. 2a). To determine the degree to which PD-L1 protein expression was dependent on LET, U2OS cells were irradiated using 10 Gy carbon-ions with LET at 13, 20, 40 or 60 keV/μm (Fig. 2b). A substantial increase in PD-L1 protein expression was observed when using LET at >13–20 keV/μm. Similar to the result of the flow cytometry experiment (Fig. 1a), PD-L1 upregulation was saturated at LET of 20–60 keV/μm although a subtle increase was observed from 20 to 60 keV/μm (Fig. 2b). A time-course experiment showed an obvious increase in PD-L1 protein expression starting at 24 h after irradiation (Fig. 2c). In this study, the RBE between X-rays and 60 keV/μm carbon-ion irradiation in U2OS cells was estimated at ~3.24 (Fig. 2d). To compare the levels of PD-L1 upregulation between X-ray and high LET carbon-ion at a similar RBE dose, cell surface PD-L1 expression was examined by flow cytometry. Interestingly, carbon-ion irradiation induced greater PD-L1 upregulation than X-rays at a similar RBE dose (Fig. 2e). These data suggest that carbon-ion-specific DNA damage, which is a complex DNA lesion, is involved in greater PD-L1 upregulation.

### ATR kinase inhibition attenuates the PD-L1 upregulation induced by high LET carbon-ion irradiation

In previous studies, we and others showed that PD-L1 expression is dependent on ATR-Chk1 signaling after X-rays [14–16]. To monitor the magnitude of ATR-Chk1 signaling after high LET carbon-ion irradiation, Chk1 phosphorylation at S345 was examined, because ATR phosphorylates Chk1 at S345 during DNA double strand break (DSB) repair. Consistent with the greater PD-L1 induction observed after high LET carbon-ion irradiation, Chk1 was more highly phosphorylated after carbon-ion irradiation than it was after X-ray irradiation (Fig. 3a). Although ATR inhibition substantially reduced Chk1 phosphorylation, we observed that ATM partially contributed to Chk1 phosphorylation after high LET carbon-ion irradiation (Fig. 3b). Similar to the result of PD-L1 upregulation, Chk1 phosphorylation seemed to be saturated after >5 Gy of 60 keV/μm. Thus, the peak of PD-L1 upregulation may be restricted by Chk1 signaling activity. This partial contribution is likely explained by the indirect activation of ATR-Chk1 signaling by ATM after IR [19, 20]. To confirm the finding that carbon-ion-induced PD-L1 upregulation is predominantly dependent on ATR kinase activity, PD-L1 expression was examined in the presence of an ATR inhibitor. ATR inhibition impaired PD-L1 upregulation after high LET carbon-ion irradiation (Fig. 3c). Moreover, flow cytometry analysis showed that ATR inhibition significantly reduced the PD-L1 upregulation, suggesting that cell surface presentation of PD-L1 after carbon-ion irradiation is dependent on ATR-Chk1 signaling (Fig. 3d).

### STAT1 phosphorylation and IRF1 expression are more significantly induced by high LET carbon-ion irradiation than they are by X-ray irradiation

**Fig. 4.**
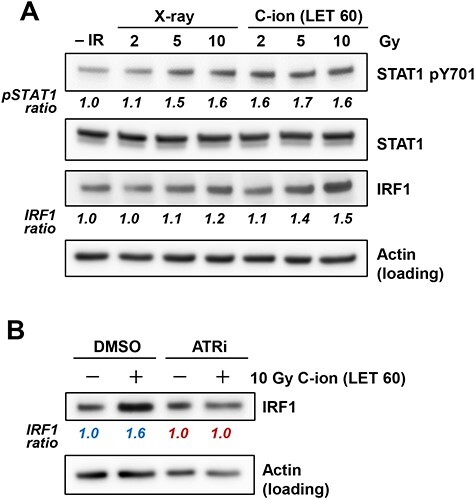
High LET carbon-ion irradiation upregulates phosphorylated STAT1 and IRF1 expression. (A) Phosphorylated STAT1 and IRF1 expression levels were examined in U2OS cells at 48 h after irradiation with 2, 5 and 10 Gy of X-rays or carbon-ion with LET at 60 keV/μm. The signal intensities of STAT1 pY701 and STAT1 were measured using ImageJ. The STAT1 pY701 signal was normalized to that of STAT1; subsequently, the ratio of STAT1 pY701 upregulation was normalized to that detected in non-irradiated cells. (B) IRF1 expression in U2OS cells was examined in the presence of 10 μM ATR inhibitor (VE821) at 48 h after irradiation with 10 Gy of carbon-ion with LET at 60 keV/μm. The signal intensities of IRF1 and actin were measured using ImageJ. The IRF1 signal was normalized to that of actin; subsequently, the ratio of IRF1 upregulation was normalized to that detected in non-irradiated DMSO (blue) or ATRi (red) cells.

DNA damage signaling upregulates STAT1 phosphorylation and IRF1 expression, transcription factors, that regulate PD-L1 expression [15, 21]. Therefore, we examined the levels of STAT1 phosphorylation and IRF1 expression after carbon-ion irradiation. STAT1 phosphorylation and IRF1 upregulation were induced in a dose-dependent manner after X-ray. In contrast, carbon-ion irradiation induced greater STAT1 phosphorylation and IRF1 expression compared with the same physical dose of X-rays (Fig. 4a). Next, we sought to examine the ATR dependency of IRF1 expression after high LET carbon-ion irradiation. Treatment with an ATR inhibitor significantly reduced IRF1 upregulation (Fig. 4b).

### Super-resolution analyses reveal increased PD-L1 presentation after high LET carbon-ion irradiation

**Fig. 5.**
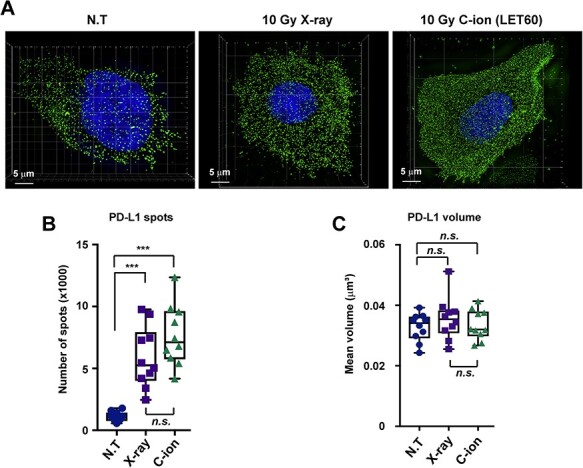
Super-resolution analyses reveal increased PD-L1 presentation after high LET carbon-ion irradiation. (A) The number of PD-L1 spots on the surface of U2OS cells was examined at 48 h after irradiation with 10 Gy of X-rays or carbon-ions with LET at 60 keV/μm. Representative images of cells after no treatment (N.T.), 10 Gy X-ray irradiation, or 10 Gy carbon-ion irradiation with LET at 60 keV/μm. Green, PD-L1; blue, DAPI. (B, C) The number of PD-L1 spots and their mean volumes on cell surfaces depicted in Fig. 5a were quantified. Statistical significance was tested by comparison with the N.T. sample. The statistical significance following Bonferroni’s correction is shown. ^***^P < 0.0005. *n.s.* not significant.

Our flow cytometry and immunoblotting analysis showed great induction of PD-L1 after carbon-ion irradiation compared with that after X-ray irradiation. However, these approaches did not clarify how PD-L1 is presented on the cell surface. Therefore, to visualize PD-L1 presentation on the cell surface, we performed an immunofluorescence-based super-resolution analysis using 3D structured illumination (3D-SIM). For immunofluorescence staining of cell surface proteins, an anti-PD-L1 antibody was incubated with living cells to minimalize the incorporation of the primary antibody inside the cell membrane. After incubation of living cells with the primary antibody, the cells were fixed to retain the anti-PD-L1 antibody on the cell surface, followed by staining with the secondary antibody and DAPI. Successful 3D-SIM imaging revealed that PD-L1 spot signals were evenly distributed on cell surfaces (Fig. 5a). This staining techniques allowed the visualization of a predominant and abundant localization of PD-L1 on cell surfaces (other angles of 3D images are shown in Supplementary Fig. 1–Fig. 3), with approximately 1000 spots observed in non-irradiated U2OS cells. In agreement with our immunoblotting and flow cytometry analyses, carbon-ion irradiation substantially increased the number of PD-L1 spots, with a greater effect than that observed after X-ray irradiation although the difference between X-ray and carbon-ion was not statistically significant (Fig. 5b). Although the number of spots was increased after irradiation, the mean volume of the PD-L1 signal remained unchanged (Fig. 5c), suggesting that PD-L1 molecules at each spot are not gathered or dissociated in response to IR.

## DISCUSSION

In this study, we investigated the responsiveness and the signal cascade of PD-L1 expression in U2OS cells after high LET carbon-ion irradiation. To examine the effects of LET on PD-L1 upregulation, we used carbon-ion irradiation with LET at 13, 20, 40 and 60 keV/μm. These LETs cover the range used in clinical settings [22]. We found that high LET carbon-ion irradiation induced higher PD-L1 expression than that observed after the same physical doses of X-ray irradiation, although the magnitude of this enhancement was close to saturation at 20–60 keV/μm, particularly at the cell surface level. Consistent with the dependency on ATR observed after X-ray irradiation [15], the upregulation of PD-L1 after high LET carbon-ion irradiation was also dependent on ATR signaling in U2OS cells. In addition, we found that the LET-dependent increase in Chk1 phosphorylation was caused by ATR in this condition. Moreover, we showed that the transcriptional regulators STAT1 and IRF1 were highly upregulated after high LET carbon-ion irradiation and that IRF1 upregulation was ATR dependent. Finally, the immunofluorescence-based super-resolution analyses revealed that the number of PD-L1 spots on cell surfaces was increased after high-LET carbon-ion irradiation compared with X-ray irradiation. Collectively, these data suggest that high LET carbon-ion upregulates PD-L1 via the same ATR-Chk1-STAT1/IRF1 pathway in U2OS cells as that observed after X-ray irradiation; however, high LET carbon-ion irradiation produces significantly greater induction of PD-L1 compared with that observed after exposure to X-rays when the same physical dose was applied.

DSBs are repaired through non-homologous end joining or homologous recombination (HR) processes [23]. During the S/G2 cell-cycle phase, some DSBs are resected by DNA nucleases to generate 3′ single-stranded DNA (ssDNA) and promote HR [23, 24]. The ssDNA on the resected DSB ends is then bound with the replication protein A (RPA); this ssDNA–RPA complex activates ATR, which then phosphorylates Chk1 [25]. The phosphorylated Chk1 further activates downstream signaling. In the G2 phase in particular, ~30% of DSBs undergo resection and are repaired by HR after X-ray irradiation [20]. However, previous studies, including our research, have shown that DSBs that are induced by high LET particle irradiation preferentially undergo HR by upregulating DSB end resection activities [19, 20]. Consistent with these molecular insights, ATR-Chk1 signaling after high LET particle irradiation is more highly upregulated by ssDNA after resection than it is after exposure to X-rays [19]. Our data also confirmed that Chk1 phosphorylation after carbon-ion irradiation is substantially greater than after X-ray irradiation in U2OS cells (Fig. 3a). Our previous study suggested that the ATR-Chk1 cascade is a central signal transducer for PD-L1 after X-ray irradiation, similar to the mechanism observed after other types of DNA-damaging exposure [15, 16]. Interestingly, PD-L1 upregulation was almost saturated at the time points (24–48 h after IR) examined in this study. This may be associated with the saturation signal of Chk1 signaling after the application of a high dose, for example, most of Chk1 proteins may be phosphorylated in the presence of a high amount of DNA damage; however, the precise underlying mechanism is unclear. Although the peak of PD-L1 upregulation is saturated, the upregulation may be sustained for a longer time after high LET or high doses of radiation. This will be addressed in future works. Importantly, our data show that high LET carbon-ion caused higher PD-L1 expression than that caused by X-ray at similar RBE doses. This can be explained by greater ATR–Chk1 activation due to hyper resection induced by complex DNA lesions after high LET particle irradiation [20]. Thus, we propose that the upregulation of PD-L1 in U2OS cells after IR is mediated via an ATR-Chk1 signal at the resected DSB ends that show a greater contribution after high LET carbon-ion irradiation.

The cGAS/STING pathway is considered as another regulatory pathway underlying PD-L1 upregulation in response to DNA damage [26, 27]. After DNA damage, cells are arrested at cell-cycle checkpoints, such as G1/S, intra-S and G2/M [23]. However, if cells harboring DSBs progress into the M phase, DNA fragments are generated during mitosis, and these micronuclear fragments are released from the primary nuclei and are detected by cGAS/STING complexes [28–30]. Several studies have shown that micronuclei (or cytosolic DNA)-dependent cGAS/STING activation transduces IFNα/β signaling in cancer cells, resulting in the upregulation of PD-L1 in tumor microenvironments [26]. Conversely, RIG-I contributes to the activation of IFNα/β signaling in normal cells [31]. A previous study showed that the cGAS/STING pathway is downregulated in U2OS cells [18]. Whether ATR-Chk1, cGAS/STING, RIG-I or other pathways coordinately contribute to PD-L1 upregulation after carbon-ion irradiation in other cell lines remains unclear; this coordination will be examined in future work.

Radiotherapy has advanced considerably with the introduction of novel technologies, such as carbon-ion radiotherapy, the clinical promise of which has been well documented in solid tumors [1, 22, 32]. The present data showed that carbon-ion irradiation substantially induced PD-L1 upregulation after high LET irradiation within the range of clinical settings. Therefore, after carbon-ion radiotherapy, the immune activity in patients may be downregulated because of increases in PD-L1 expression in the tumor environment. Thus, although carbon-ion radiotherapy has a strong cell-killing effect against solid tumors, the radiation-dependent immune activation may not have been fully boosted. To overcome this issue, anti-PD1/PD-L1 therapy would be of benefit together with or following carbon-ion radiotherapy, so that suppressed immune activity can be normalized and tumors, including metastases, may be eradicated.

## Supplementary Material

Supplemental_materials_rrab050
