# Supplementary material for: High linear energy transfer carbon-ion irradiation upregulates PD-L1 expression more significantly than X-rays in human osteosarcoma U2OS cells
Source: J Radiat Res. 2021 Jul 1;62(5):773–81. doi: 10.1093/jrr/rrab050 (PMC8438258; doi:10.1093/jrr/rrab050)
Supplement: Supplemental_materials_rrab050 [file supplemental_materials_rrab050.pdf]

Supplementary Figure 1. No treatment

Top view

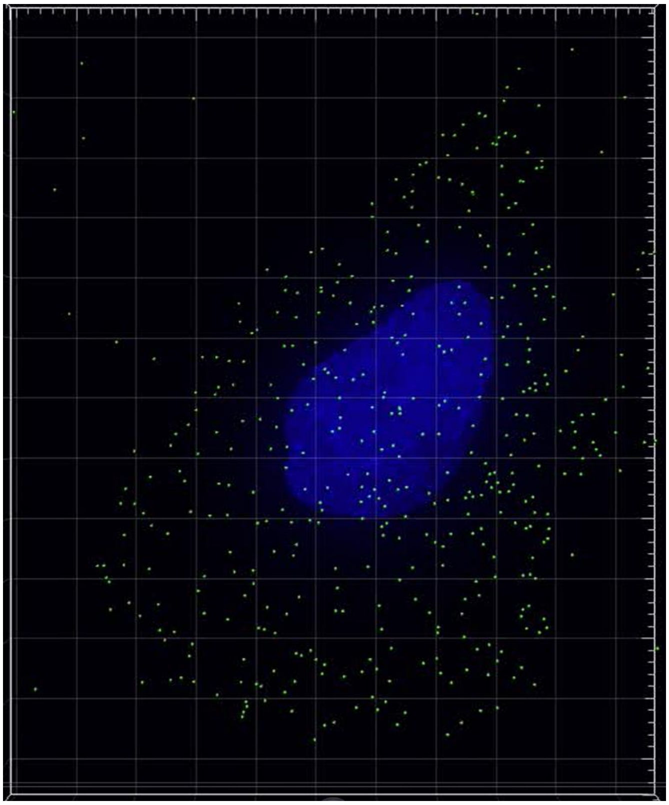

Angle view

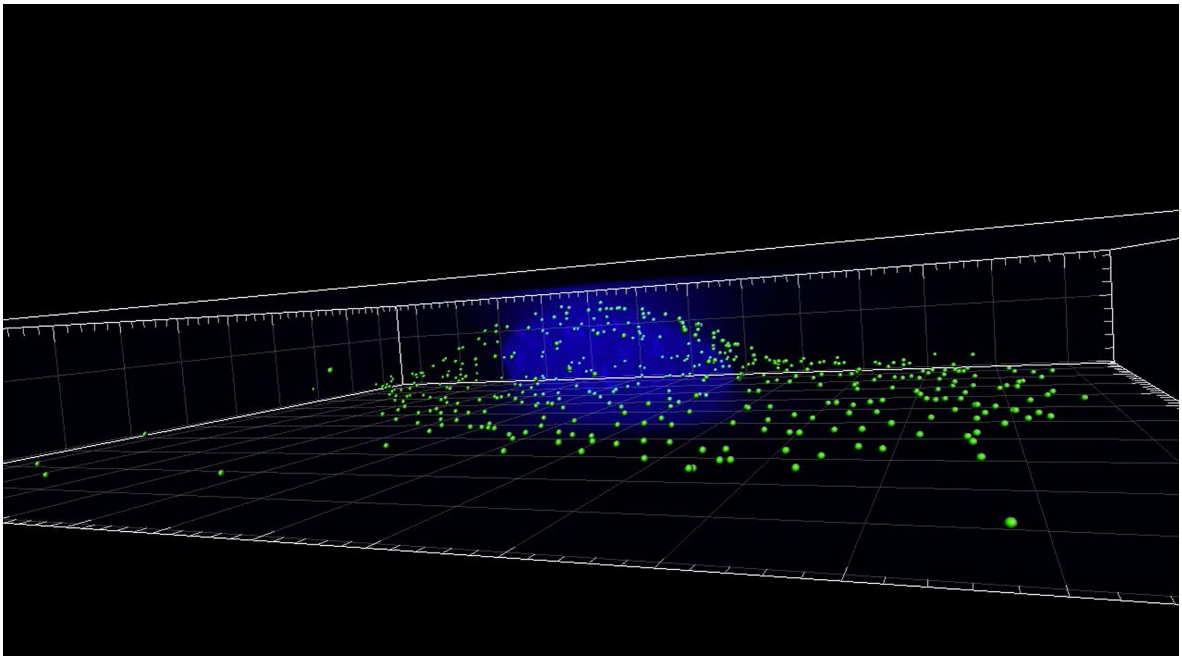

Supplementary Figure 2. 10 Gy X-ray

Top view

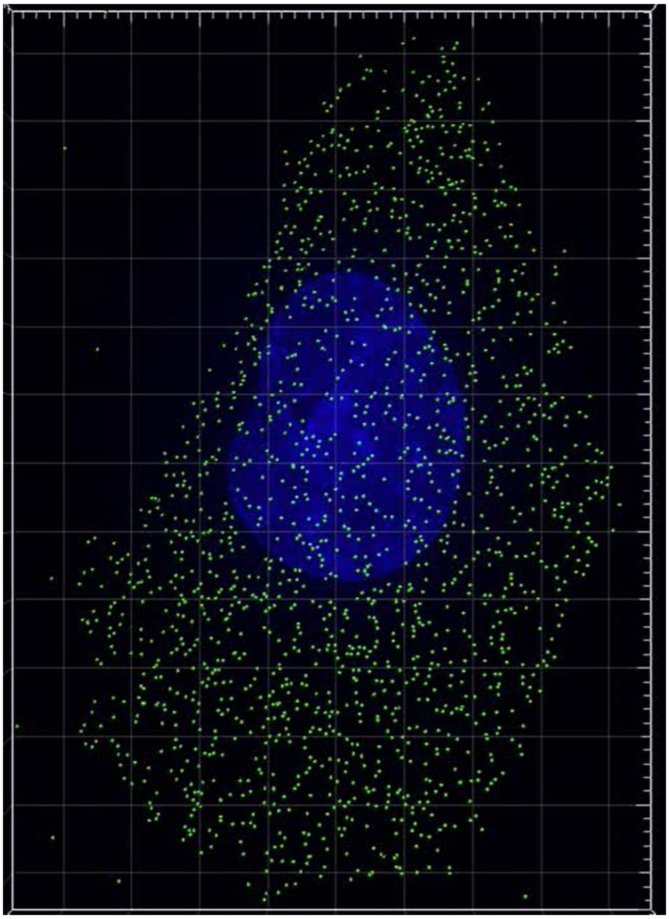

Angle view

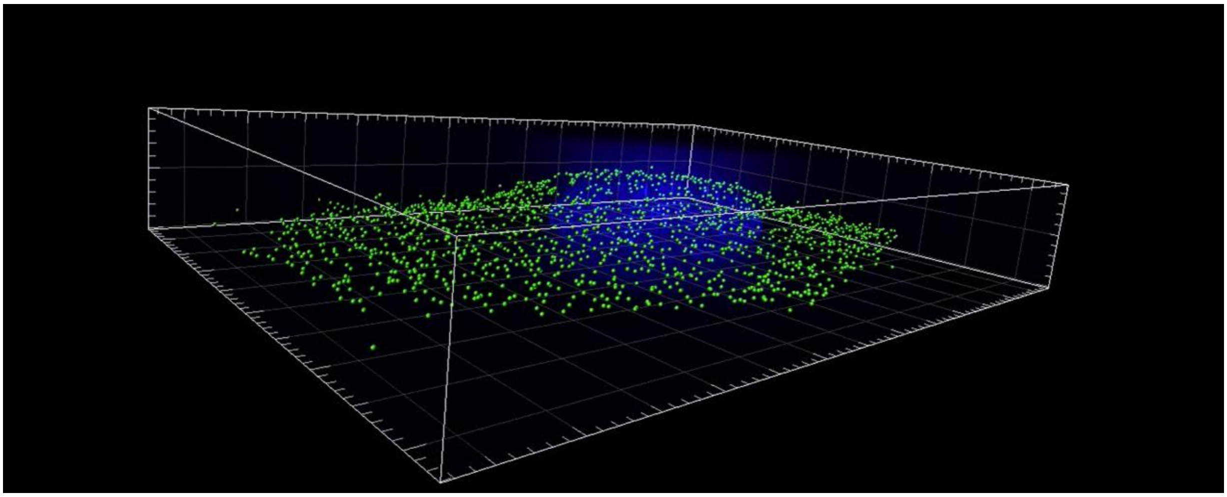

Supplementary Figure 3

Top view

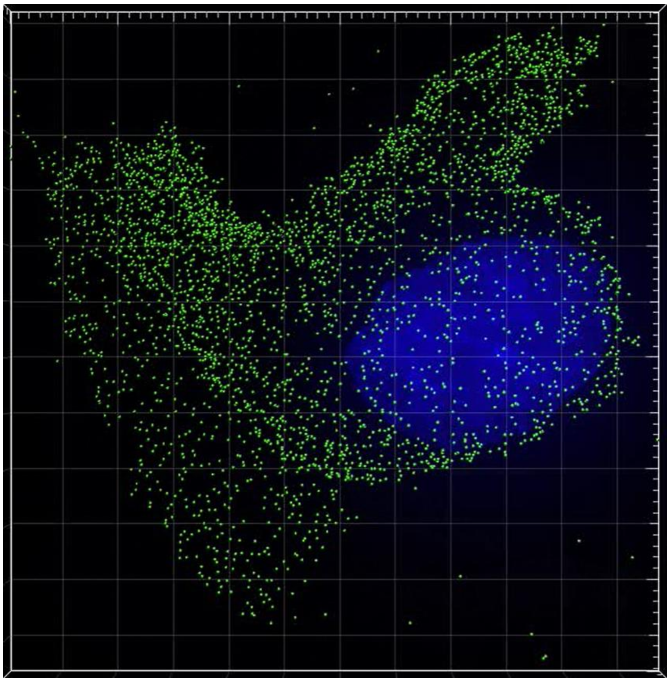

Angle view

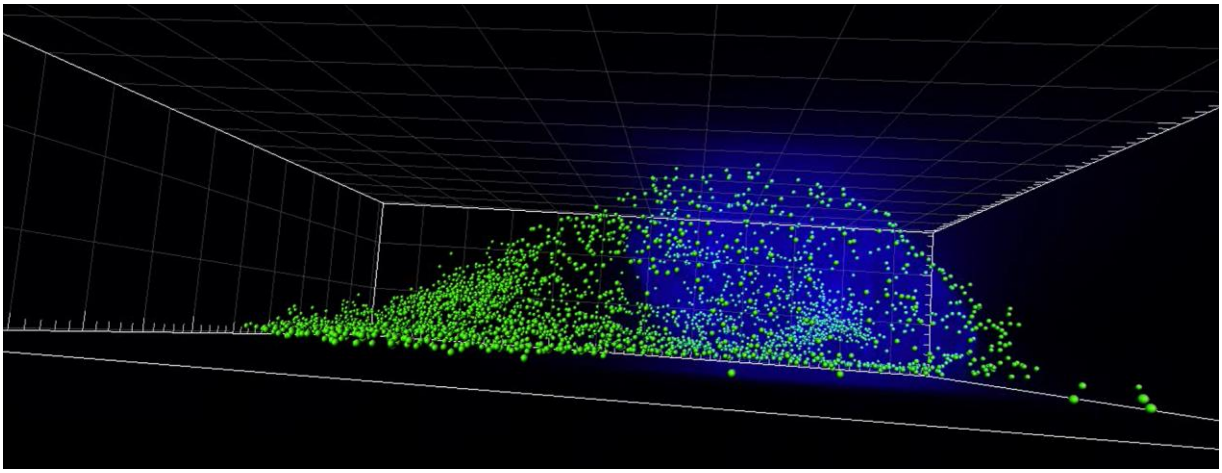

Supplementary Figures

Representative images of cell surface PD-L1 expression in U2OS cells are shown. The distribution of PD-L1 signal was identified using the Spots mode. Green, PD-L1; Blue, DAPI.

Supplementary Figure 1 No treatment

Supplementary Figure 2 10 Gy X-ray irradiation 48 h

Supplementary Figure 3 10 Gy carbon-ion irradiation with LET at 60 keV/μm 48 h

Supplementary Table 1: List of antibodies used in the present study.

Target	Mono/ polyclonal	Clone/ reference	Antibody raised in	Source	Dilution for I.B. or I.F.
Actin	Mono	8H10D0	Mouse	Cell Signaling Tech.	1:10,000 (I.B.)
Chk1	Mono	2G1D5	Mouse	Cell Signaling Tech.	1:500 (I.B.)
pChk1	Mono	133D3	Rabbit	Cell Signaling Tech.	1:500 (I.B.)
IRF1	Mono	D5E4	Rabbit	Cell Signaling Tech.	1:1000 (I.B.)
PARP-1	Poly	9542	Rabbit	Cell Signaling Tech.	1:1000 (I.B.)
PD-L1	Mono	E1L3N	Rabbit	Cell Signaling Tech.	1:1000 (I.B.)<br>1:100 (I.F.)
STAT1	Mono	D1K9Y	Rabbit	Cell Signaling Tech.	1:1000 (I.B.)
STAT1 pY701	Mono	58D6	Rabbit	Cell Signaling Tech.	1:200 (I.B.)

I.B., Immunoblotting; I.F., Immunofluorescence

Supplementary Table 2: List of antibodies used for flow cytometry in the present study.

Antibody information for FACS	Source
APC Mouse IgG2b, κ Isotype Ctrl	Biolegend
APC anti-human CD274 (B7-H1, PD-L1), (Mouse IgG1, κ)	Biolegend
